# Personality characteristics of a sample of violent adolescents against their partners

**DOI:** 10.1186/s41155-019-0122-7

**Published:** 2019-04-15

**Authors:** María Penado Abilleira, María Luisa Rodicio-García, Tania Corrás Vázquez, María Paula Ríos de Deus, María Josefa Iglesias Cortizas

**Affiliations:** 1 Universidad Isabel I de Castilla, Burgos, Spain; 2 Universidad de A Coruña, Coruña, Spain; 3 Pontevedra, Spain

**Keywords:** Dating violence, Personality, Anger, Frustration, Adolescents

## Abstract

**Background:**

The study of intimate partner violence has historically focused on violence perpetrated on females by males, but recent research suggests that, at least in teenage couples, the difference between genders is decreasing or even reversing. The objective of this study is to analyze the personality characteristics of adolescents who are violent with their partners. The sample consisted of 430 subjects (229 girls and 201 boys), between 14 and 19 years (*M* = 16.18, SD = 1.81), middle or high school students, which completed the Personality Assessment Inventory-Adolescents and the Conflict in Adolescent Dating Relationships Inventory.

**Results:**

The results show that girls have higher personality scores on the scales that show problems of internal behavior (depression and anxiety), while boys show higher scores on the scales of external behavior problems (antisocial behavior and drug use). Through a regression analysis, the results show predictive weights in the aggression traits (*β* = .331, *p* < .001), antisocial characteristics (*β* = .202, *p* < .001), and mania (*β* = .185, *p* < .05), as the scores on the scale of violence perpetrated increase in girls. For boys, personality variables do not seem to have such a decisive weight to explain the violence committed, since only heat and alcohol problems represent 5.4% of the variance found. These differences between boys and girls should be analyzed in future studies and, if the findings are maintained, taken into account when developing programs to prevent gender-based violence in adolescents.

**Conclusions:**

The results of this study show how the personality characteristics have a differential weight in the explanation of the teen dating aggression according to the gender of the aggressors, with a greater relevance in the prediction of the aggressive behaviors committed by the girls.

## Background

The Centers for Disease Control and Prevention (2016) defines teen dating violence as physical, sexual, psychological, or emotional violence, as well as stalking, within a dating relationship. It can take place in person or electronically and might occur between a current or former dating partner. Since the first studies done by Makepeace nearly 40 years ago, attention has been drawn to the phenomenon of abusive interactions that occur among young couples involved in intimate relationships, termed “teen dating violence” (Ortega, Ortega-Rivera, & Sánchez, 2008; Pazos, Oliva, & Hernando, 2014). Previous research carried out has focused on analyzing the prevalence of the phenomenon (Fernández-Fuertes, Orgaz, & Fuertes, 2011; Foshee et al., 2011; Muñoz-Rivas, Graña, O’Leary, & González, 2009), risk factors associated with the perpetrators (Rey-Anaconda, 2008; Rubio-Garay, Carrasco, Amor, & López-González, 2015), and the psychological after-effects exhibited by some of the victims who are usually minors (Carrascosa, Cava, & Buelga, 2016; Exner-Cortens, Eckenrode, & Rothman, 2013). Even though, the study of intimate partner violence has historically focused on violence perpetrated by males over females (Ramirez & Nuñez, 2010; Vázquez & Castro, 2008), recent research suggests that, at least in teenage couples, the difference between genders is decreasing or even reversing (Taylor & Mumford, 2014).

This role reversal, which implies the equal use of violence within intimate relationships by men and women, occurs independent of the cultural context, with a growing number of studies that indicate the phenomenon is spreading on a global scale. Sears, Byers, and Price (2007) state that in the USA, 43% of teenage boys and 51% of teenage girls between 12 and 18 years have physically, psychologically, or sexually abused their partners. Males reported having used forms of psychological violence in 35% of the cases, with 15% of cases involving physical violence and 17% involving sexual violence. In the case of females, 47% reported having used forms of psychological violence, with 28% of cases involving physical violence and 5% involving sexual violence. In this scenario, with the exception of sexual violence, females reported having perpetrated the same form of abusive behaviors more often than their male counterparts.

In the most recent examination of teen dating violence carried out in Spain (Rubio-Garay, López-González, Carrasco, & Amor, 2017), researchers claim that between 7.7 and 40.3% of physical violence is perpetrated by males and between 3.8% and 41.9% is perpetrated by females. The data show that psychological violence is the most common type of violence between men and woman with a wide range of occurrence (4–97%). Finally, sexual violence perpetrated by males against females ranges from 2.6 to 58.8%, while for females against males, the range is less frequent (between 1.2 and 40.1%). In Latin American populations, Rivera-Rivera, Allen-Leigh, Rodríguez-Ortega, Chávez-Ayala, and Lazcano-Ponce (2007) found that 4.2% of females and 4.3% of males had engaged in psychological violence and that 21% of females and 19.5% of males had reported having engaged in acts of physical violence. Drawing from the data obtained from a sample of over 7000 Mexican students between 12 and 24 years, the authors concluded that females reported being the perpetrators of violence in the relationship to a greater degree. In the same context, but with Colombian population, Rey-Anaconda, Mateus-Cubides, and Bayona-Arévalo (2010) found that 41.7% of males and 58.3% of females between 15 and 20 years had engaged in some form of abusive behavior against their partner at least once.

Within the personality variables that can explain teen dating violence, high scores are observed in general distress, negative affect, anxiety, and impulsivity for boys and girls who commit acts of violence against their partner (Boivin, Lavoie, Hebert, & Gagne, 2012; Gover, Kaukinen, & Fox, 2008; Moore, Elkins, McNulty, Kivisto, & Handsel, 2011; Wolfe, Wekerle, Scott, Straatman, & Grasley, 2004). Regarding the personality variables that differentiate them, depressive symptoms and other internalizing symptoms are the ones that most correlate with the gender violence committed by girls (Banyard, Cross, & Modecki, 2006; Chase, Treboux, & O’Leary, 2002; Foshee, Linder, MacDougall, & Bangdiwala, 2001), in addition to hostility and anger (Boivin et al., 2012; Wolfe et al., 2004). In the case of boys, the differentiating personality variables found behind the commission of acts of violence against their partner would be low empathy, low self-esteem (Brendgen, Vitaro, Tremblay, & Wanner, 2002; Hanby, Fales, Nangle, Serwik, & Hedrich, 2012; Renner & Whitney, 2012; Wolfe et al., 2004), and antisocial behaviors (Gamez-Gaudix, Straus, & Hershberger, 2011; Monson & Langhinrichsen-Rohling, 2002; Moore et al., 2011).

Finally, the relationship between alcohol and drug use does not seem to follow a differential pattern according to gender, with studies that indicate the absence of association between these variables and the commission of aggressive acts against the couple (Foshee, McNaughton Reyes, & Ennett, 2010; Hammock & O’Hearn, 2002), while others affirm that this association occurs independently of gender, being this variable predictive of gender violence for boys and girls (Baker & Stith, 2008; Banyard et al., 2006; Foshee et al., 2001; Foshee et al., 2010; Gamez-Gaudix et al., 2011; Luthra & Gidycz, 2006; O’Keefe, 1997; Shook, Gerrity, Jurich, & Segrist, 2000). In summary, while some personality variables seem to explain gender violence among adolescents without differentiating the gender of the aggressor (substance abuse), others are clearly differentiated, with a higher score on antisocial behavior for boys who commit acts of violence against their partners and high scores in depression and internal problems for girls who are violent against their partner.

## Method

### Aim

Based on previous results that show a pattern of differential behavior of personality variables (antisocial behavior, depression, and internal problems) in boys and girls who are violent against their partner, in addition to following the recommendations of authors who suggest continuing to perform differentiating analyses according to gender to better understand the predictive variables of gender violence among adolescents (Dardis, Dixon, Edwards, & Turchik, 2015), the following objectives are pursued by this research:

1. To study the prevalence of aggressive behaviors against the couple in a sample of adolescents according to gender
2. Identify the differential personality variables of boys and girls
3. Analyze which personality variables predict violence against the couple in adolescent boys and girls

### Participants and research design

This study is based on a sample of 616 students (boys and girls) in their final years of middle school and junior and senior high school, from four of the six public high schools in Pontevedra (Galicia, Spain). The parents of all under 18 adolescents were informed of the tasks to be carried out and were provided with informed consent with the objectives of the investigation to authorize access to their children which was collected on the day of the intervention. All subjects and their parents gave written informed consent after receiving a comprehensive description of the study protocol, and there was no family or any student who rejected our invitation. Participants had volunteered to be involved in this study, and they were not given any incentive to take part in it.

On the survey administration day, researchers informed all participants about the objectives of the study and reassured them of the anonymity of the data. They furthermore emphasized that their participation was voluntary and that they had the right to withdraw from the study at any time; none of the students withdrew their participation. The questionnaire was administered during school hours (during the study hours that students have in their school hours and with an application time that had not exceeded 60 min), under the supervision of the researchers in charge of the study. The sampling was non-probabilistic, casual, or accidental, since it was attended by students who were in the classroom at the time of the application. The researchers underlined the importance of providing individual responses and emphasized that the behaviors gathered in the scale were a serious matter, and not a game, so those who wished to participate were encouraged to give honest answers.

In addition to providing their sociodemographic data, they provided responses to the Personality Assessment Inventory-Adolescents (PAI-A) (Morey, 2007) and the Conflict in Adolescent Dating Relationships Inventory (CADRI) (Wolfe et al., 2001). Since one of the conditions was that they had to be in an intimate relationship at the present time, or had to have been in one during the last 12 months, and having carried out gender violence behaviors at some point in their relationship, teenagers that fulfilled this condition in the CADRI (Wolfe et al., 2001) were selected. As a result, 177 participants (98 girls and 79 boys) who had not been in a relationship in the last 12 months were eliminated for failing to fulfill the above requirement. Finally, of the 439 adolescents who fulfilled the above conditions, nine were discarded (seven girls and two boys) because their responses in the PAI-A (Morey, 2007) showed that they had not paid attention to the items when responding (inconsistency) or they responded randomly (infrequently) and therefore were eliminated because they did not comply with the validity scales. After the sample data was purged, statistical analyses were carried out on the results obtained from 430 adolescents (229 girls and 201 boys), between 14 and 19 years (*M* = 16.18, SD = 1.81). All couples participating in the study were maintaining a heterosexual relationship.

The heads of the academic institutions were contacted to request the institutions’ participation. Information was also collected as a part of the city council’s efforts to raise awareness about gender violence among teens. The parents of all minors were informed as to the tasks that would be performed. Once their approval was obtained, an informed consent form was distributed to underage students so that it could be completed by their parents to provide consent for their children to participate in the study. On the same day as the request, the staff in charge informed the participants of all ages of the study’s objectives and the anonymous nature of the data, stressing that their participation in the study was voluntary and informing them of their right to withdraw their participation. The questionnaires, administered by the staff in charge of the study, were filled out in a group format and during school hours for each of the grades.

This study was approved by the Ethics Committee of the University of Coruña (Spain). Data were processed in compliance with the Spanish Data Protection Law. All subjects gave written informed consent in accordance with the Declaration of Helsinki.

### Measures

#### Conflict in Adolescent Dating Relationships Inventory (CADRI)

The estimation of intimate partner violence among adolescents was obtained using the committed violence subscale of the CADRI (Wolfe et al., 2001), adapted to the Spanish population by Fernández-Fuertes, Fuertes, and Pulido (2006). This inventory comprises 35 dual-nature items (referring to the behavior performed and the behavior suffered), 10 of which do not assess violent behavior to serve as distractions (“I discussed the issue calmly”, “I gave reasons for my side of the argument”). The 25 remaining items assess the violent behavior perpetrated and suffered based on five dimensions, which include sexual abuse (“I touched him/her sexually when she/he didn’t want me to”), relational abuse (“I tried to turn his/her friends against him/her”), verbal/emotional abuse (“I spoke to him/her in a hostile or mean tone of voice”), threatening behavior (“I threatened to hurt him/her”), and physical abuse (“I slapped him or pulled his hair”). Participants must rate each statement on a 4-point Likert scale based on its frequency of occurrence in the relationship over the last year, where never (this has never happened in our relationship) is 0, seldom (has only happened once or twice) is 1, sometimes (has happened between 3 and 5 times) is 2, and often (has happened 6 or more times) is 3. This tool is reliable in both its original scale (*α* = .83) and its adaptation (*α* = .85). In the sample obtained, the reliability results are even higher than in the Spanish adaptation, with Cronbach’s alpha coefficient being .918 for the total scale, and slightly lower for the perpetrated violence subscale (*α* = .860). For the factors that form the subscale of violence committed, the reliability results show good results when we analyze the factor of verbal-emotional abuse (*α* = .839) and physical abuse (*α* = .747), while the reliability is lower when considering the factor of sexual abuse (*α* = .427), relational abuse (*α* = .399), and threatening behavior (*α* = .394).

#### Personality Assessment Inventory-Adolescent (PAI-A)

The PAI-A (Morey, 2007) was used to obtain personality measurements, adapted to the Spanish population by Cardenal, Ortiz-Tallo, and Santamaría (2012). Adolescents must assess how the inventory’s 264 statements reflect their way of being, thinking, feeling, and acting, on a scale ranging from False (0), Slightly True (1), Mainly True (2), and Very True (3). The results provide a comprehensive assessment of the adolescent’s psychopathology, measured on a 22-point scale: 4 validity scales (inconsistency, infrequency, negative impression, and positive impression), 11 clinical scales (somatic concerns, anxiety, anxiety-related disorders, depression, mania, paranoia, schizophrenia, borderline features, antisocial features, alcohol problems, and drug problems), 5 treatment consideration scales (aggression, suicidal ideation, nonsupport, stress, and treatment rejection), and 2 interpersonal scales (dominance and warmth).

The reliability data obtained in the assessment of the instrument with the Spanish population show good results for all scales considered, with Cronbach’s alpha coefficient ranging from .79 to .92 for the clinical scales, .73 to .96 for the treatment consideration scales, and .65 to .77 for the interpersonal scales. The reliability results obtained in the sample of adolescents corroborate the validity of the instrument, with Cronbach’s alpha coefficient for the global sample of .946 (.942 when we focus on boys and .945 in the case of girls).

### Data analysis

The data was analyzed using SPSS v.22.0 statistical software. Initially, Pearson’s correlation analysis was used between the perpetrated violence subscale of the CADRI and the clinical scales, the treatment consideration scales and the interpersonal scales of PAI-A. To get an estimation of the influence of personality variables in explaining violence perpetrated by teenage girls, a stepwise regression analysis was carried out, taking the perpetrated violence scale as the dependent variable, and the clinical scales, treatment consideration scales, and interpersonal scales as independent or explanatory variables.

## Results

The results show different percentages of prevalence depending on the type of violence committed by boys and girls. It is interesting to note that the behaviors most frequently claimed by females are the verbal-emotional ones (90.40% of the girls vs. 82.6% of the boys) and physical abuse (24.02% of the girls vs. 7.46% of the boys), which involve doing something to make their partners jealous, digging up something bad that they had done in the past, saying something to make them angry, speaking in a hostile or offensive tone of voice, insulting or humiliating their partner, and hitting and throwing an object. In the case of the boys, scores are slightly lower than those obtained by the girls, but with a similar prevalence of each type of violence and where it is observed that they report a lower prevalence of verbal-emotional abuse behaviors but they admit to carrying out sexual abuse behaviors more frequently than the girls (50.75% compared to 31.87% of the girls) (see Table 1).

**Table 1:** Adolescents who committed violent acts against their partner by gender

	Boys		Girls		*p*	*eta*
	*n*	%	*n*	%		
Sexual abuse	102	50.75	73	31.87	.001**	.616
Relational abuse	17	8.46	11	4.8	.082	.378
Threatening behavior	27	13.44	47	20.52	.004**	.586
Physical abuse	15	7.46	55	24.02	.000**	.659
Verbal-emotional abuse	166	82.6	207	90.40	.001**	.709

**p < .01

Despite the high number of adolescents who admit to having committed acts of violence at some point in their relationship, the frequency of such behaviors can be considered low, judging by the average scores obtained. In this sense, it is observed how all the aggressive behaviors recognized by adolescents have happened once or twice in the last year (rarely) which would indicate that it is sporadic or not continuous behaviors. An analysis based on gender indicates that it is women who present more frequently physical abuse (*t* = − 2.861, g.l. = 420, *p* = .000 < .01) and verbal emotional aggression (*t* = − 2.712, g.l. = 419, *p* = .001 < .01) than boys, while it is these who most frequently engage in sexual violence behaviors than girls (*t* = .693, g.l. = 427, *p* = .002 < .01) (see Table 2).

**Table 2:** Means and standard deviations for each of the factors of violence committed

	Boys		Girls		*p*
	*M*	SD	*M*	SD	
Sexual abuse	1.57	.34	1.33	.19	.002**
Relational abuse	1.40	.13	1.48	.31	.354
Threatening behavior	1.26	.06	1.27	.10	.415
Physical abuse	1.25	.10	1.65	.45	.000**
Verbal-emotional abuse	1.58	.39	1.73	.50	.001**

**p < .01

Regarding the results obtained in the PAI-A raw scores, the girls in the sample show greater internal problems related to anxiety (*t* = − 9.201, g.l. = 428, *p* = .000 < .05), anxiety-related disorders (*t* = − 7.385, g.l. = 428, *p* = .000 < .05), depression (*t* = − 5.097; g.l. = 428; *p* = .000 < .05), somatization (*t* = − 4.961, g.l. = 428, *p* = .000 < .05), paranoia (*t* = − 4.409, g.l. = 428, *p* = .000 < .05), schizophrenia (*t* = − 2.260, g.l. = 428, *p* = .024 < .05), and borderline features (*t* = − 7.435, g.l. = 428, *p* = .000 < .05) than the sample of boys. In the case of boys, it is the external behavioral problems that seem to have a higher incidence, with a high score in antisocial behavior (*t* = 3.564, g.l. = 428, *p* = .000 < .05), problems with drugs (*t* = 1.599, g.l. = 428, *p* = .040 < .05), aggression (*t* = 2.746, g.l. = 428, *p* = .006 < .05), and rejection of treatment (*t* = 6.251, g.l. = 428, *p* = .000 < .05) (see Table 3).

**Table 3:** Means and standard deviations for each of the PAI scales (raw scores)

	Boys				Girls				*p*
	Min	Max	*M*	SD	Min	max	*M*	SD	
Clinical scales									
SOM	0	38	7.97	6.56	0	44	10.84	7.27	.000**
ANX	0	43	15.70	8.14	2	48	22.30	9	.000**
ARD	2	35	14.75	6.82	3	38	19.12	7.36	.000**
DEP	1	42	13.81	7.85	0	49	17.39	8.94	.000**
MAN	0	38	19	7.34	2	44	18.35	7.60	.292
PAR	5	48	18.19	7.75	1	42	20.90	7.14	.000**
SCZ	0	35	11.13	7	0	42	12.46	7.12	.024*
BOR	1	48	18.18	9.60	6	53	24.08	9.53	.000**
ANT	0	39	14.06	7.34	0	37	11.95	7	.000**
ALC	0	19	2.69	3.30	0	22	3.12	3.52	.138
DRG	0	24	3.12	4.12	0	21	2.92	3.87	.040*
Treatment consideration scales									
AGG	1	41	15.89	7.34	1	54	11.95	7	.006**
SUI	0	24	2.29	4.19	0	24	2.94	4.83	.089
STR	0	16	2.33	3.17	0	17	3.57	3.76	.060
NON	0	18	4.87	3.67	0	18	4.56	4.07	.340
RXR	0	18	11.58	3.72	1	18	9.70	3.56	.000**
Interpersonal scales									
DOM	3	24	12.90	3.81	2	23	12.35	4	.093
WRM	3	24	14.23	4.10	3	24	14.73	3.88	.135

*p < .05; **p < .01

To rule out the existence of collinearity in the associations between the study variables and to explore the significance and direction of these associations, a matrix of correlations was made between the violence committed subscale of the CADRI and the clinical scales, the treatment consideration scales, and the interpersonal scales of PAI-A. The correlation analyses carried out for the girls show significant correlations among the clinical, treatment consideration, and interpersonal scales. Within the clinical scales, there were significant associations between perpetrated violence and antisocial features (*r* = .403, *p* < .01), mania (*r* = .379, *p* < .01), borderline features (*r* = .345, *p* < .01), drug problems (*r* = .270, *p* < .01), alcohol problems (*r* = .238, *p* < .01), and paranoia (*r* = .230, *p* < .01). Heightened scores in the abovementioned scales are associated with people who have problems with authority; who are egocentric; lack empathy and stability (antisocial features); who have high levels of activity, irritability, and impatience (mania); who have unstable and fluctuating interpersonal relationships and difficulty controlling anger (borderline features); who may present problems controlling their consumption of drugs (drug problems) and alcohol (alcohol problems); and who tend to feel resentful toward or hold grudges against the people around them (paranoia).

For the treatment consideration scales, there are significant correlations between the perpetrated violence scale and the aggression scale (*r* = .468, *p* < .01), which are associated with people who have issues related to anger, assertiveness, hostility, and aggression. We also observed a significant negative correlation with treatment rejection as a treatment consideration scale (*r* = − .212, *p* < .01), which suggests the person has little interest in making personal psychological or emotional changes. Finally, within the interpersonal scales, there is a significant correlation between the perpetrated violence scale and the dominance scale (*r* = .222, *p* < .01), which reveals a dominant style in the subject’s interpersonal relationships (see Table 4). In the case of boys, the correlations are significant only in the clinical scale that measures alcohol problems (*r* = .195, *p* < .05) and in the interpersonal scale that measures warmth (*r* = .196, *p* < .05). The high scores in the previous scales describe an individual with problems in the consumption of alcohol or with negative consequences derived from the consumption, but with a sociable, understanding, and pleasant personality.

**Table 4:** Pearson’s correlation between CADRI’s committed violence subscale and PAI-A clinical, treatment and interpersonal scales for girls (below diagonal) and boys (above diagonal)

	1	2	3	4	5	6	7	8	9	10	11	12	13	14	15	16	17	18	19
1. CCS	1	.030	.076	.147	− .072	.125	.020	.045	.047	.056	.195*	.072	.102	− .103	.065	− .096	− .102	.069	.196*
2. SOM	.127	1	.602**	.580**	.577**	.276**	.542**	.585**	.593**	.274**	.270**	.139*	.265**	.406**	.506**	.336**	− .523**	.018	− .026
3. ANX	.124	.575^**^	1	.701**	.670**	.255**	.559**	.664**	.731**	.156*	.191**	.118	.354**	.340**	.518**	.312**	− .648**	− .112	− .046
4. ARD	.08	.456^**^	.675^**^	1	.579**	.311**	.502**	.588**	.653**	.042	.106	.014	.169**	.332**	.518**	.279**	− .573**	− 083	.043
5. DEP	.061	.500^**^	.556^**^	.443^**^	1	.099	.641**	.728**	.703**	.265**	.283**	.161**	.313**	.549**	.644**	.594**	− .620**	− .186**	− .208**
6. MAN	.379^**^	.222^**^	.286^**^	.341^**^	− .128	1	.349**	.324**	.448**	.244**	.109	.083	.250**	.163**	.229**	.001	− .398**	.329**	.363**
7. PAR	.230^**^	.248^**^	.303^**^	.340^**^	.434^**^	.275^**^	1	.643**	.681**	.333**	.245**	.201**	.432**	.388**	.503**	.424**	− .513**	.038	− .139*
8. SCZ	.112	.406^**^	.507^**^	.458^**^	.480^**^	.356^**^	.390^**^	1	.696**	.292**	.259**	.110	.298**	.432**	.570**	.489**	− .631**	− .034	− .117
9. BOR	.345^**^	.525^**^	.661^**^	.560^**^	.530^**^	.418^**^	.541^**^	.573^**^	1	.389**	.306**	.272**	.555**	.480**	.599**	.399**	− .680**	− .009	.039
10. ANT	.403^**^	.128	.071	.042	.077	.418^**^	.251^**^	.259^**^	.314^**^	1	.387**	.453**	.546**	.239**	.182**	.196**	− .281**	.214**	.039
11. ALC	.238^**^	.179^*^	.123	.075	.051	.177^*^	.057	.103	.227^**^	.372^**^	1	.490**	.312**	.242**	.165**	.1	− .262**	.066	.101
12. DRG	.270^**^	.203^*^	.11	.012	.108	.174^*^	.064	.121	.255^**^	.433^**^	.388^**^	1	.315**	.263**	.124*	.128	− .180**	.013	.057
13. AGG	.468^**^	.136	.162^*^	.044	.115	.332^**^	.361^**^	.139	.503^**^	.372^**^	.221^**^	.363^**^	1	.265**	.199**	.157	− .232**	.169**	− .131*
14. SUI	.078	.431^**^	.344^**^	.276^**^	.578^**^	− .005	.337^**^	.466^**^	.482^**^	.048	.072	.159^*^	.206^*^	1	.388**	.482^**^	− .291**	− .041	− .105
15. STR	.171^*^	.519^**^	.390^**^	.414^**^	.605^**^	.161^*^	.451^**^	.386^**^	.556^**^	.233^**^	.116	.194^*^	.226^**^	.497^**^	1	.682^**^	− .571**	− .093	− .023
16. NON	− .018	.345^**^	.226^**^	.180^*^	.596^**^	0	.403^**^	.357^**^	.400^**^	.115	.1	.128	.157	.482^**^	.682^**^	1	− .318**	− .188**	− .410**
17. RXR	− .212^**^	− .504^**^	− .634^**^	− .512^**^	− .566^**^	− .234^**^	− .388^**^	− .513^**^	− .673^**^	− .147	− .105	− .133	− .132	− .348^**^	− .478^**^	− .406^**^	1	.024	− .082
18. DOM	.222^**^	− .174^*^	− .182^*^	− .049	− .378^**^	.341^**^	− .063	− .295^**^	− .064	.153	.001	.136	.288^**^	− .220^**^	− .125	− .249^**^	.181^*^	1	.250**
19. WRM	.036	− .047	.149	.168^*^	− .155	.260^**^	− .064	− .214^**^	.045	.094	.097	− .041	.026	− .214^**^	− .095	− .275^**^	.012	.323^**^	1

*CCS* CADRI committed subscale, *SOM* somatic concerns, *ANX* anxiety, *ARD* anxiety-related disorders, *DEP* depression, *MAN* mania, *PAR* paranoia, *SCZ* schizophrenia, *BOR* borderline features, *ANT* antisocial features, *ALC* alcohol problems, *DRG* drug problems, *AGC* aggression, *SUI* suicidal ideation, *NON* non-support, *STR* stress, *RXR* treatment rejection, *DOM* dominance, *WRM* warmth

*p < .05 **p < .01

In order to identify the proportion of variance explained by the personality variables in the violence committed subscale, a multiple linear regression analysis was carried out taking as predictors, or independent variables, all the values obtained by the subjects in PAI-A clinical (somatic complaints, anxiety, anxiety-related disorders, depression, mania, paranoia, schizophrenia, limit traits, antisocial traits, alcohol problems, and drug problems), treatment (aggression, suicidal idea, stress, lack of social support, and refusal to treatment), and interpersonal scales (dominance and warmth). The dependent variable was the violence committed subscale measured through the CADRI. For each of the regression analyses, an examination of the behavior of the data was carried out with the purpose of identifying extreme multivariate cases; that is, those products of relations between variables that constitute values that depart considerably from the behavior of the whole. It was considered important to detect these values to ensure an adequate adjustment of the regression solution to the general trend of the data. For this analysis of multivariate extreme cases, a Mahalanobis distance and Cook distance were calculated. In no case was a Mahalanobis distance greater than the critical value (*p* = .001 < .05), and Cook’s distance was .05, which indicates that, even if the case were eliminated, there would not be a significant change in the regression coefficients. The analysis of the residues shows that there are no outliers, since the maximum and minimum values of the typified residues are less than 3 in absolute value.

The results demonstrate how three overall scales explain 29.3% of the variability found in the perpetrated violence scale by girls (aggression, antisocial features, and mania). Of the three previous variables, aggression considerably increases the predictive value of the model, with a 21.4% increase in explanatory power. From the above results, we can state that aggression (*t* = 4.448, *p* < .001), antisocial features (*t* = 2.620, *p* < .001), and mania (*t* = 2.429, *p* < .05) contribute to explaining the model, with a greater predictive weight for aggression (*β* = .331, *p* < .001) and a similar predictive weight for antisocial features (*β* = .202, *p* < .001) and mania (*β* = .185, *p* < .05) (see Tables 5 and 6).

**Table 5:** General statistics of the regression model between CADRI committed subscale and PAI-A global scales (girls)

Step	Predictors	*R*	*R* ^2^	Adjusted *R*^2^	Unstandardized coefficients		Standardized coefficients	*t*	Sig.
					*B*	Std. error	Beta		
1	AGC	.468^a^	.219	.214	0.309	.069	.331	4.448	.000
2	ANT	.529^b^	.279	.270	0.211	.080	.202	2.620	.000
3	MAN	.553^c^	.306	.293	0.137	.056	.185	2.429	.016

^a^Predictors: (constant), aggression

^b^Predictors: (constant), aggression, antisocial features

^c^Predictors: (constant), aggression, antisocial features, mania

**Table 6:** Variables excluded from the regression analysis (girls)

Variables	Beta	*t*	Sig.	Partial correlation	Colinearity statistics
					Tolerance
SOM	− .076	− 1.223	.223	− .083	.932
ANX	.029	.452	.652	.031	.895
ARD	.049	.773	.441	.052	.892
DEP	− .004	− .069	.945	− .005	.936
PAR	− .019	− .285	.776	− .019	.799
SCZ	− .080	− 1.228	.221	− .083	.862
BOR	.010	.132	.895	.009	.695
ALC	.059	.919	.359	.062	.896
DRG	.067	1.041	.299	.070	.879
SUI	− .065	− 1.047	.296	− .071	.943
STR	− .028	− .443	.658	− .030	.944
NON	− .115	− 1.867	.063	− .125	.959
RXR	− .042	− .664	.507	− .045	.908
DOM	.053	.808	.420	.055	.859
WRM	− .025	− .406	.685	− .027	.954

*CCS* CADRI committed subscale, *SOM* somatic concerns, *ANX* anxiety, *ARD* anxiety-related disorders, *DEP* depression, *MAN* mania, *PAR* paranoia, *SCZ* schizophrenia, *BOR* borderline features, *ANT* antisocial features, *ALC* alcohol problems, *DRG* drug problems, *AGC* aggression, *SUI* suicidal ideation, *NON* non-support, *STR* stress, *RXR* treatment rejection, *DOM* dominance, *WRM* warmth

Based on the previous results, and taking into account the subscales that make up each of the global scales of the PAI-A, the personality variables that explain female violence show us girls with an increase in activity, with a decrease in dream and accelerated pattern, with excessive self-esteem and overvaluation of one’s own ideas, and are impatient and demanding with others (lack of empathy). These are adolescents with a predisposition to verbally show anger, with shouting or an insulting language, who believe in the use of violence as a behavioral strategy. Regarding the antisocial behavior, young adolescent girls that perform violent acts against their partner show egocentricity and high narcissism with tendencies to look for strong emotions and low tolerance to frustration.

In the case of the boys, the personality variables do not seem to be after the explanation of the variability found in the subscale of violence committed since only two characteristics (warmth and alcohol problems) would explain only 5.4% of the variance, with some predictive weights lower than those found for the girls (see Tables 7 and 8).

**Table 7:** General statistics of the regression model between CADRI committed subscale and PAI-A global scales (boys)

Step	Predictors	*R*	*R* ^2^	Adjusted *R*^2^	Unstandardized coefficients		Standardized coefficients	*t*	Sig.
					*B*	Std. error	Beta		
1	WRM	.196^a^	.038	.031	.090	.044	.176	2.053	.042
2	ALC	.262^b^	.069	.054	.078	.039	.175	2.034	.044

^a^Predictors: (constant), warmth

^b^Predictors: (constant), warmth, alcohol problems

**Table 8:** Variables excluded from the regression analysis (boys)

Variables	Beta	*t*	Sig.	Partial correlation	Colinearity statistics
					Tolerance
SOM	− .023	− .257	.798	− .023	.930
ANS	.036	.409	.683	.036	.957
ARD	.118	1.375	.172	.121	.986
DEP	− .107	− 1.152	.252	− .102	.844
MAN	.039	.421	.675	.037	.854
PAR	− .009	− .101	.920	− .009	.873
SCZ	− .007	− .074	.941	− .007	.892
BOR	− .020	− .228	.820	− .020	.904
ANT	− .001	− .011	.991	− .001	.878
DRG	.003	.027	.979	.002	.828
AGC	.111	1.200	.232	.106	.842
SUI	− .155	− 1.726	.087	− .151	.891
STR	.037	.435	.665	.039	.986
NON	− .046	− .474	.637	− .042	.792
RXR	− .044	− .498	.619	− .044	.930
DOM	.017	.190	.849	.017	.959

*CCS* CADRI committed subscale, *SOM* somatic concerns, *ANX* anxiety, *ARD* anxiety-related disorders, *DEP* depression, *MAN* mania, *PAR* paranoia, *SCZ* schizophrenia, *BOR* borderline features, *ANT* antisocial features, *ALC* alcohol problems, *DRG* drug problems, *AGC* aggression, *SUI* suicidal ideation, *NON* non-support, *STR* stress, *RXR* treatment rejection, *DOM* dominance, *WRM* warmth

## Discussion

The results obtained suggest that females who use violence in their intimate relationships are characterized by the use of verbal-emotional and physical aggression, in addition to having low frustration tolerance and a lack of empathy in personal relationships. The use of verbal-emotional and physical aggression by girls confirms the results found in previous research (Sears et al., 2007; Pazos et al., 2014; Vicario-Molina, Orgaz Baz, Fuertes Martín, González Ortega, & Martínez Álvarez, 2015; Rubio-Garay et al., 2017). Focusing on the results obtained in the boys’ sample, a higher prevalence of sexual violence behaviors is observed, as has been pointed out in other studies up to now (Rubio-Garay et al., 2017; Sears et al., 2007). These differences in the prevalence of aggressive behaviors based on gender are consistent with those found by other studies conducted in the same population which is the sample object of this study (Spanish adolescents), and where there is a greater presence of aggressive physical and psychological (emotional) behavior among girls while a greater frequency of violent sexual behavior among boys (Sebastian, Vergudo, & Ortiz, 2014).

The differences in prevalence observed in terms of gender, with a higher percentage of girls who acknowledge exercising physical violence against their partner, are consistent with the results obtained by other authors and where it has been pointed out that when the presence of physical violence was measured by self-reports, as is our case, it was more frequent in women than in men (White, 2009). The greater frequency of aggressive behavior in young girls can be explained equally by the legitimization of aggressive behaviors by boys, which minimize their aggressive behaviors and downplay them, while girls perform an overvaluation of their actions and they feel guilty for it (González-Ortega, Echeburúa, & Corral, 2008).

In general, the high percentage of adolescents who claim to have violence against their partner is confirmed by previous studies that affirm the increase in this type of behavior and that argue that the use of violence by adolescents has become a way to solve conflicts within couple relationships (González & Santana, 2001; González-Ortega et al., 2008; Muñoz-Rivas, Graña, O’Leary, & González, 2007). Authors argue that many adolescents consider aggression as something inherent in the relationship (Avery-Leaf, Cascardi, O’Leary, & Cano, 1997), minimizing them and even denying them, especially when sporadic (Arriaga, 2002). Thus, physical aggression (e.g., slapping, hitting, or punching) as a way to solve conflicts is considered a “normal” practice by many couples (Hird, 2000).

In the studied sample of adolescents, a differential pattern according to gender in the personality variables is also observed. Girls have greater internal behavior problems (anxiety, anxiety-related disorders, depression, somatization, paranoia, schizophrenia, and borderline features) while in the case of boys it is the external behavioral problems that seem to have a higher incidence (antisocial behavior, problems with drugs, and aggression) (Muñoz-Rivas, Gámez-Guadix, Graña & Fernández, 2010). The previous personality characteristics are similar to those observed in previous studies, where a higher incidence of antisocial behavior is observed in adolescent boys (Lanctôt, 2015; López & Rodríguez-Arias, 2010; Ma, 2005) and higher rates of depression and anxiety in girls (McLaughlin & King, 2015).

Prediction analyses point to different behaviors in boys and girls in terms of what personality variables are behind the violent behavior against the couple. Thus, in the case of girls, they are aggression, antisocial features, and mania, aggression being the one that presents a greater predictive power. In the case of boys, the personality variables do not seem to be behind the explanation of the variability found in the violence committed, since only two characteristics (warmth and alcohol problems) enter the explanatory model and with a very low percentage load. The weight of antisocial behavior and the mania traits found in the sample of girls is similar to that found by other authors who have studied the behavior of interpersonal violence (IPV) in adult women, where they observed high scores of these two constructs (Capaldi, Knoble, Shortt, & Kim, 2012; Goldenson, Geffner, Foster, & Clipson, 2007; Hughes, Stuart, Gordon, & Moore, 2007; McKeown, 2014). In the case of the boys, in addition to the small weight obtained by the personality variables in the explanation of the gender violence committed, it is observed that this violence is explained, as indicated by previous studies, by the consumption of alcohol (Muñoz-Rivas, Gámez-Guadix, Graña, & Fernández, 2010), being a novelty until the moment the introduction of the warmth construct within the variables that explain the commission of acts of gender violence.

The information on predominant personality characteristics in each subject, boy or girl, will make it possible to act at different levels: developing coping strategies for conflict situations associated with each type of personality; enhancing the necessary skills for a harmonious coexistence; informing and educating about gender roles and their applicability into dating relationships; launching programs to promote conflict resolution skills, emotional regulation, social skills, and communication; and, ultimately, developing a culture of prevention and early intervention that diminishes and eradicates gender violence in the relationship of adolescent couples.

Before concluding, it is important to keep in mind the limitations of this study. Firstly, the results were obtained from a sample of adolescents who are currently in a heterosexual relationship, or have been in the last 12 months and, therefore, the conclusions cannot be generalized to describe other populations. Secondly, the transversal and descriptive nature of the study makes it difficult to establish causal relationships between the personality variables and the violence committed, since the changes that occur may vary over time. Finally, despite insisting that the participants contribute sincere affirmations, the social desirability present in subjects of this age group should not be ignored, which could moderate the results obtained, this being one of the reasons that can explain the significance in the character trait, warmth, found in the sample of boys.

## Conclusions

The results of this study show that teen-dating aggression is a reality when it is verified that almost a third of the adolescents studied have shown some type of violent behavior against their partner in the last year. Within these violent behaviors, the most frequent are those considered verbal-emotional type. Considering the gender of the aggressor, it is the boys who make the most use of sexual violence, while the girls admit to carrying out more aggressive physical behaviors.

The study of the personality characteristics associated with these acts of violence indicates a differential pattern according to gender and where a greater weight of this type of variables is observed for adolescent girls. Thus, personality characteristics such as aggression, egocentricity, narcissism, and low tolerance to frustration predict the aggressive behaviors recognized by girls. In the case of boys, the personality variables studied have a low predictive value, which would indicate that other types of variables, out of the scope of this study, and may explain this type of violence (stereotypes, cultural patterns, gender roles, etc.)

## Abbreviations

CADRI: Conflict in Adolescent Dating Relationships Inventory
PAI-A: Adolescent Personality Assessment Inventory-Adolescents
